# Alcohol consumption and risk of psoriasis: Results from observational and genetic analyses in more than 100,000 individuals from the Danish general population

**DOI:** 10.1016/j.jdin.2024.03.003

**Published:** 2024-03-20

**Authors:** Alexander Jordan, Charlotte Näslund-Koch, Signe Vedel-Krogh, Stig Egil Bojesen, Lone Skov

**Affiliations:** a Department of Dermatology and Allergy, Copenhagen University Hospital - Herlev and Gentofte, Copenhagen, Denmark; b Department of Clinical Medicine, Faculty of Health and Medical Sciences, University of Copenhagen, Copenhagen, Denmark; c Department of Clinical Biochemistry, Copenhagen University Hospital - Herlev and Gentofte, Copenhagen, Denmark; d Copenhagen General Population Study, Copenhagen University Hospital - Herlev and Gentofte, Copenhagen, Denmark; e Copenhagen City Heart Study, Copenhagen University Hospital - Herlev and Gentofte, Copenhagen, Denmark

**Keywords:** *ADH1B*, *ADH1C*, alcohol, epidemiology, Mendelian randomization, psoriasis, risk factors

## Abstract

**Background:**

Psoriasis is associated with high alcohol consumption, but the causality of this relationship is unclear.

**Objective:**

We aimed to use a Mendelian randomization approach to investigate the causal effects of alcohol on incident psoriasis.

**Methods:**

We included 102,655 adults from the prospective Copenhagen studies. All participants filled out a questionnaire on alcohol consumption, were physically examined, and had blood drawn for biochemical and genetic analyses. We created a genetic instrument based on the number of fast-metabolizing alleles in alcohol dehydrogenase 1B and alcohol dehydrogenase 1C, known to be associated with alcohol consumption, to test whether alcohol consumption was causally associated with psoriasis.

**Results:**

Observationally, we found an increased risk of incident psoriasis among individuals with high alcohol consumption compared to those with low alcohol consumption with a hazard ratio of 1.30 (95% confidence interval 1.05-1.60) in the fully adjusted model. Using genetic data to predict alcohol consumption to avoid confounding and reverse causation, we found no association between number of fast-metabolizing alleles and risk of psoriasis.

**Limitations:**

Alcohol consumption was self-reported and psoriasis was defined using the International Classification of Diseases 10th revision and 8th revision codes.

**Conclusion:**

Alcohol consumption is observationally but not causally associated with incident psoriasis.

**Capsule Summary**

• Alcohol consumption is known to be associated with psoriasis, but it is unknown if this association is causal or caused by confounding or reverse causation.
• We found no evidence that alcohol consumption is causally related to psoriasis. Avoidance of excessive alcohol consumption is still recommended to reduce alcohol-related comorbidities.

## Introduction

Psoriasis is a common chronic inflammatory skin disease causing a substantial health burden for patients and society.^1^ The disease is associated with a range of comorbidities and lifestyle factors such as dyslipidemia,^2^ hypertension,^3^^,^^4^ obesity,^5^ diabetes,^6^ and smoking.^7^ Psoriasis has also been associated with higher alcohol consumption in multiple studies.^8^, ^9^, ^10^ However, in 2013 a systematic review concluded that even though alcohol consumption was higher in patients with psoriasis compared to healthy controls, there was insufficient evidence to determine if alcohol was a causal risk factor for psoriasis.^11^ Subsequently, a large prospective cohort study did not find increased risk of incident psoriasis with increasing alcohol consumption after adjustment for potential confounders.^12^ These studies are based on observational data, which comes with the inherent limitations of reverse causation and confounding factors.^13^ The ideal way of examining the causal association between alcohol consumption and psoriasis would be to conduct a randomized controlled trial with alcohol as the intervention. This is neither feasible nor ethical; however, there is an epidemiological method that can be used in these situations. Mendelian randomization (MR) relies on the fact that genetic variants are randomly assigned at conception, which can be thought of as a quasirandom natural experiment.,^14^ circumventing many sources of bias.^15^ In MR studies, these randomly assorted genetic variants are used as instruments of the exposure itself (such as alcohol). In this way, confounders are evenly distributed between groups and since genes are present from birth, the method is not prone to reverse causation.^14^

The presence of fast-metabolizing variants of the genes alcohol dehydrogenase 1B (*ADH1B*) and alcohol dehydrogenase 1C (*ADH1C*) have been linked to decreased alcohol consumption compared to the slow-metabolizing variants in several distinct populations.^16^, ^17^, ^18^, ^19^, ^20^ The genes encode 2 isoforms of the enzyme ADH which facilitates the conversion of ethanol to acetaldehyde in the main pathway for metabolism of alcohol.^17^^,^^21^ Acetaldehyde is toxic and causes unpleasant effects such as nausea, vomiting, flushing, and headache.^22^ The main hypothesis for why these genetic variants affect alcohol consumption is that fast metabolism of alcohol leads to greater accumulation of acetaldehyde and thus, more pronounced side effects in individuals with fast-metabolizing variants.^23^ The genes of *ADH1B* and *ADH1C* have been used as genetic instruments for alcohol consumption in previous MR studies.^20^^,^^24^

In this study, we hypothesized that high alcohol consumption is both observationally and causally associated with psoriasis and tested this using a MR approach in >100,000 individuals from the Danish general population.

## Methods

### Study cohort

The study cohort was formed by individuals from the 2 Danish prospective general population studies; the Copenhagen General Population Study (CGPS) and the Copenhagen City Heart Study (CCHS). For both studies a subset of Danish citizens aged 20-100 years living in the Copenhagen were invited, with a participation rate of 43% to 74%.^18^^,^^25^ The CCHS was initiated in 1976, with follow-up examinations in 1981-1983, 1991-1994, and 2001-2003, in which all previous individuals were reinvited as well as a number of new individuals were invited. Only individuals from the 1991-1994 and 2001-2003 examinations were included in this study as older samples have not been genotyped. The CGPS was initiated in 2003 with continuous enrollment until 2015. During inclusion and at follow-up examinations (only CCHS), participants filled out a questionnaire about lifestyle factors and health status. In addition, a physical examination was performed including measurement of blood pressure, body mass index (BMI), and blood samples were collected for biochemical and genetic analyses. If individuals participated in multiple examinations, the earliest examination was used to provide the longest follow-up for development of psoriasis. To avoid population stratification, we only included individuals of Scandinavian descent in the present study.^26^ Furthermore, we excluded individuals who did not answer the questions about alcohol consumption. We also excluded individuals who reported no use of alcohol, as we wanted to examine the effects of alcohol consumption on the risk of psoriasis. Moreover, individuals with no use of alcohol are different from individuals with alcohol consumption as a high proportion of these have prior alcohol dependency or severe comorbidities, which could confound the results of this study.^27^^,^^28^

All individuals gave written informed consent. The study was conducted according to the Declaration of Helsinki and approved by Danish ethical committees (KF-100.2039/91 and H-KF-01-144/01).

This study was conducted and reported in accordance with the recommendations of the Strengthening the Reporting of Observational Studies in Epidemiology.^29^

### Assessment of alcohol consumption

Alcohol consumption was self-reported and included amount and type of alcohol, which was converted into grams per week (g/week) as done in previous studies.^20^^,^^30^ One unit of alcohol was defined as 10 mL or 8 g pure alcohol.^31^ For the observational analyses, we divided the individuals into 3 groups defined according to National Health Service alcohol guidelines (United Kingdom), recommending an intake of no more than 14 units per week. This recommendation is in line with the recommendation from the Danish Health Authority, recommending no more than 120 g/week.^32^ Low consumption was defined as being within National Health Service recommendations (1-112 g/week), moderate consumption as twice the recommendations (113-224 g/week), and high consumption as >224 g/week.

### *ADH1B* and *ADH1C* genotyping

DNA was isolated from full blood and stored at −45 °C. Individuals were genotyped for the *ADH1B* genotype (rs1229984; Arg47His) and *ADH1C* genotype (rs698; Ile349Val) by Nanogen Technology^33^ and TaqMan assays (Applied Biosystems). Genotypes were in Hardy-Weinberg equilibrium in both studies (*P* > .05). Genotyping was blinded to alcohol consumption and psoriasis. Reruns were performed twice and call rates were >99.8%. We modeled our genetic instrument as the number of fast-metabolizing alleles of the 2 genes. Each gene could be homozygous for the slow variant (0 fast alleles), heterozygous fast/slow (1 allele), or homozygous for the fast variant (2 alleles). As very few individuals were homozygous for *ADH1B*, these were grouped together with the heterozygotes. Thus, an individual could have 0 to 3 or more fast-metabolizing alleles in total.

### Assessment of psoriasis

Individuals with psoriasis were identified using the World Health Organization International Classification of Diseases (ICD) codes for psoriasis: ICD-8 696.09, 696.10, 696.19, and ICD-10 L40. Diagnoses were obtained by linking each individual with the Danish National Patient Registry (DNPR). The DNPR is a nationwide register containing information on all inpatients and outpatients in Danish hospitals since 1977.^34^ Patients with mild psoriasis are often only diagnosed and treated at the general practitioner or at a private practice dermatology clinic, and only referred to the hospital in case of more severe disease. Thus, using ICD-codes to identify patients with psoriasis will mainly capture patients with moderate to severe psoriasis, as previously validated.^35^^,^^36^ In addition, these diagnoses are primarily made by dermatologists, ensuring high accuracy. In sensitivity analyses, we used a more restrictive definition of psoriasis including only ICD-8 code 696.19 and ICD-10 codes L40.0 and L40.9. Information on diagnosis was collected from January 1977 to December 2018, which was the latest available update of the DNPR in CGPS and CCHS.^31^

### Statistical analysis

All analyses were conducted in R version 3.13.0. A two-sided *P* value of < .05 was considered statistically significant. Baseline variables were compared using chi-square tests for categorical variables and analysis of variance or Kruskal-Wallis tests for continuous variables depending on whether they were normally distributed. The F-statistic was calculated using analysis of variance.

We used Cox proportional hazards models to estimate risk of incident psoriasis in observational prospective analyses. Individuals were left-censored at the time of cohort entry and with age as the underlying time-scale (age-adjusted). Individuals with a diagnosis of psoriasis prior to inclusion in the study were excluded from these analyses (*n* = 525). End of follow-up was at a diagnosis of psoriasis (*N* = 1166), death (*N* = 14,402), or on the 13th of December 2018 (latest update of the DNPR), whichever came first. In the observational cross-sectional analyses, we used a logistical model, in which psoriasis was only counted if diagnosed prior to study entry. We included the following potential confounders and adjusted for them in both prospective and cross-sectional analyses: study cohort (CGPS/CCHS), year of inclusion, sex, age, smoking status (current, prior, or never smoker), low education (defined as only mandatory primary school education or lower), low income (defined as a household income less than 50% of the median income), poor mental health (defined as answering no to the question “I am well” or yes to the question “I have considered giving up”), physical inactivity in spare time (<2 h activity/week), BMI, and diabetes (use of antidiabetic medicine, blood sugar >11 mmol/L or diagnosed in the DNPR). We used both a simple model adjusted only for study cohort, sex, age, and year of inclusion as well as a fully adjusted model including all abovementioned confounders. In genetic analyses we included cases of psoriasis independent of diagnosis time and only used the simple model as genes are assigned at conception and thus not susceptible to confounding.

Data was imputed based on sex and age using multiple regressions for continuous variables and the missing indicator method for categorical variables. The data was 97% complete. We repeated the prospective analyses as a complete case analysis in which only individuals with no missing covariates were included and found similar results to those reported.

## Results

**Fig 1:**
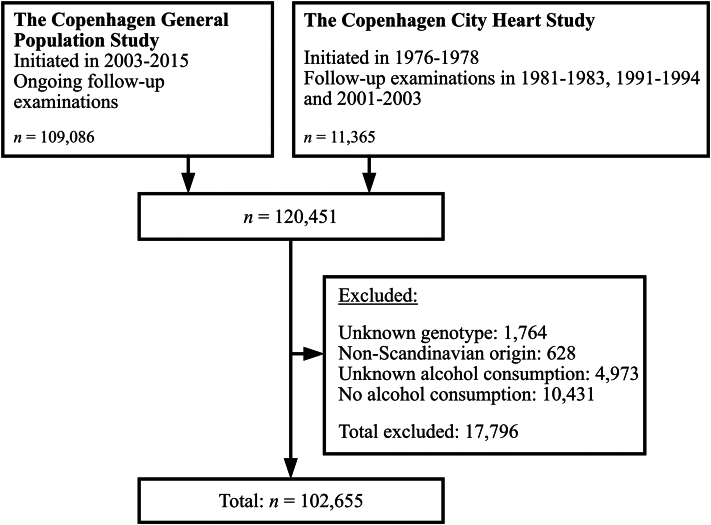
Study flow chart. *n*, Number.

A total of 102,655 individuals were included in the study (Fig 1), of which 525 (0.5%) were diagnosed with psoriasis prior to inclusion and 641 (0.6%) were diagnosed during follow-up. Baseline characteristics are shown in Table I. Compared to those with moderate and low consumption, individuals with high alcohol consumption were older, more likely to be male and smokers, had higher average BMI and higher prevalence of diabetes and hypertension. Baseline characteristics were mostly independent of fast-metabolizing allele count, apart from sex and prevalence of diabetes.

**Table I:** Baseline characteristics at inclusion according to alcohol consumption (upper section) and genotype (lower section)

	Alcohol consumption			*P* value
	1-112 g/week	113-224 g/week	>224 g/week	
Number of individuals	52,953	29,980	19,722	
Female, *n* (%)	33,939 (64.1)	14,632 (48.8)	5195 (26.3)	<.0001
Age, mean (SD), years	55.9 (13.7)	59.6 (12.6)	61.0 (11.5)	<.0001
Smoking status				<.0001
Never smoked, *n* (%)	23,486 (46.1)	10,683 (37.0)	4762 (24.9)	-
Prior smoker, *n* (%)	19,101 (37.5)	12,858 (44.5)	8960 (46.9)	-
Current smoker, *n* (%)	8380 (16.4)	5323 (18.4)	5366 (28.1)	-
Pack years, median (IQR)∗	13.5 (5.0-26.2)	15.2 (6.1-30.0)	24.0 (11.0-40.0)	<.0001
Low education, *n* (%)†	4675 (8.9)	2282 (7.6)	1533 (7.8)	<.0001
Low income, *n* (%)‡	18,006 (34.4)	9800 (33.1)	6622 (33.8)	.0005
Poor mental wellbeing, *n* (%)	6872 (13.1)	3545 (11.9)	2725 (13.9)	<.0001
Low physical activity, *n* (%)	3220 (6.1)	1483 (5.0)	1331 (6.8)	<.0001
Body mass index, median (IQR), kg/m^2^	25.2 (22.8-28.1)	25.4 (23.2-28.0)	26.1 (23.8-28.7)	<.0001
Hypertension, *n* (%)	24,316 (45.9)	16,160 (53.9)	12,572 (63.7)	<.0001
Diabetes mellitus, *n* (%)	1933 (3.7)	1111 (3.7)	937 (4.8)	<.0001

	Number of fast-metabolizing alleles				*P* value
	0	1	2	3	
Number of individuals	17,869	48,178	34,379	2229	
Female, *n* (%)	9569 (53.6)	25,262 (52.4)	17,829 (51.9)	1106 (49.6)	.0001
Age, mean (SD), years	57.8 (13.2)	58.0 (13.2)	57.9 (13.2)	58.5 (13.0)	.13
Smoking status					.59
Never smoked, *n* (%)	6754 (39.3)	18,302 (39.4)	13,075 (39.5)	800 (37.3)	-
Prior smoker, *n* (%)	7092 (41.2)	19,203 (41.3)	13,705 (41.4)	919 (42.8)	-
Current smoker, *n* (%)	3351 (19.5)	8940 (19.2)	6352 (19.2)	426 (19.9)	-
Pack years, median (IQR)∗	15.8 (6.0-30.1)	16.0 (6.2-30.9)	16.0 (6.0-30.0)	17.4 (6.0-32.0)	.23
Low education, *n* (%)†	1498 (8.4)	4068 (8.5)	2757 (8.0)	167 (7.5)	.08
Low income, *n* (%)‡	6016 (34.1)	16,228 (34.0)	11,463 (33.7)	721 (32.7)	.49
Poor mental wellbeing, *n* (%)	2301 (13.0)	6169 (12.9)	4383 (12.8)	289 (13.1)	.97
Low physical activity, *n* (%)	1096 (6.2)	2806 (5.9)	2012 (5.9)	120 (5.4)	.33
Body mass index, median (IQR), kg/m^2^	25.5 (23.1-28.2)	25.5 (23.1-28.2)	25.4 (23.1-28.2)	25.3 (23.0-28.0)	.06
Hypertension, *n* (%)	9245 (51.7)	24,963 (51.8)	17,704 (51.5)	1136 (51.0)	.73
Diabetes mellitus, *n* (%)	720 (4.0)	1944 (4.0)	1250 (3.6)	67 (3.0)	.003

∗ Among current or prior smokers.

† Having no secondary education.

‡ Household income <50% of the median.

### Observational analysis

**Fig 2:**
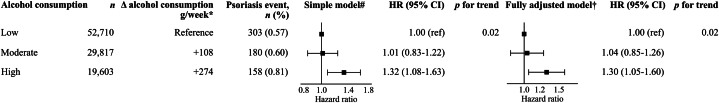
Observational analysis. Risk of incident psoriasis was estimated using Cox proportional hazards. 525 individuals with psoriasis prior to baseline were excluded from this analysis. ∗Geometric mean difference compared with the group before. #Adjusted for sex, age, study cohort and year of inclusion. †Adjusted for sex, age, study cohort, year of inclusion, smoking status, low education, and low income, poor mental health, physical inactivity, body mass index and diabetes. *CI*, Confidence interval; *HR*, hazard ratio; *n*, number.

In the prospective analyses, individuals with a high alcohol consumption had an increased risk of incident psoriasis with a hazard ratio of 1.30 (95% CI 1.05-1.60, *P* = .01) in the fully adjusted analysis (Fig 2). Moderate versus low alcohol consumption was not associated with psoriasis (1.04, 0.85-1.26). However, there was a significant trend across the 3 alcohol groups (*P* = .02). Similar results were seen in the cross-sectional analyses estimating risk of prevalent psoriasis according to alcohol consumption (Supplementary Fig 1, available via Mendeley at https://data.mendeley.com/datasets/xv2x88cd5g/1).

For sensitivity, we used a more restrictive definition of psoriasis (ICD-8: 696.19 and ICD-10: L40.0 and L40.9) and only including individuals with full information on covariates, which both provided similar results (Supplementary Fig 2, available via Mendeley at https://data.mendeley.com/datasets/xv2x88cd5g/1).

Furthermore, we conducted stratified analyses for sex, BMI, and age and found no interaction between alcohol consumption and the mentioned covariates on risk of incident psoriasis (Supplementary Fig 3, available via Mendeley at https://data.mendeley.com/datasets/xv2x88cd5g/1).

### Genetic analysis

**Fig 3:**
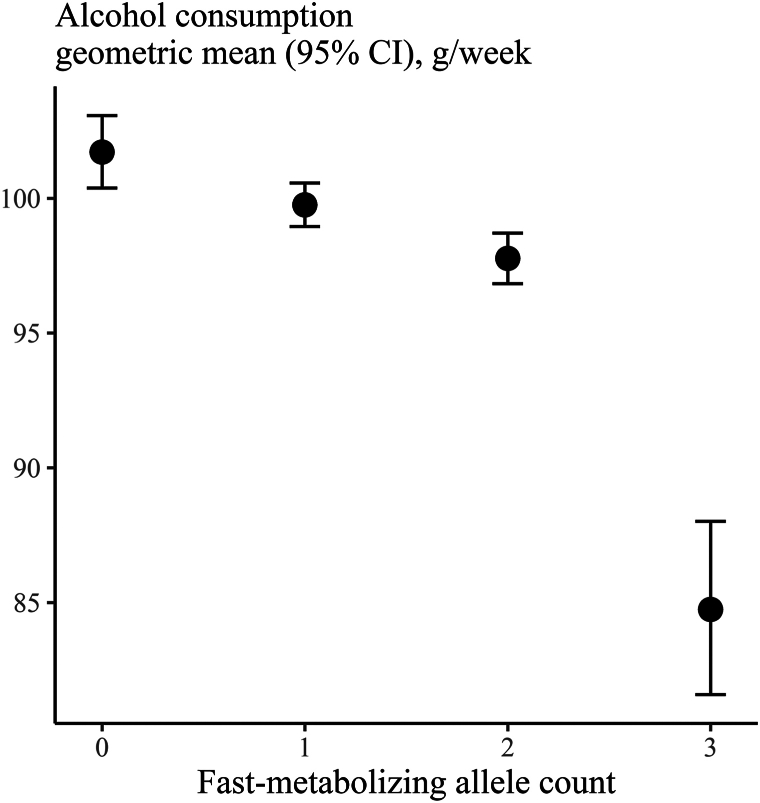
Alcohol consumption by number of fast-metabolizing alleles (0, 1, 2, or 3) in the genes *ADH1B* and *ADH1C*. Error bars represent 95% confidence intervals. *CI*, Confidence interval.

As expected, alcohol consumption decreased with increasing number of fast-metabolizing alleles (Fig 3). On average, individuals with 3 or more fast-metabolizing alleles had a decrease in alcohol consumption of 17 g/week (95% CI 20-13, *P* < .0001), equivalent to approximately 1.5 standard drinks in the United States, compared to those with zero fast-metabolizing alleles. The combined *ADH1B* and *ADH1C* genotype was considered an adequate genetic instrument, with an F-statistic of 38.4.^26^

**Fig 4:**
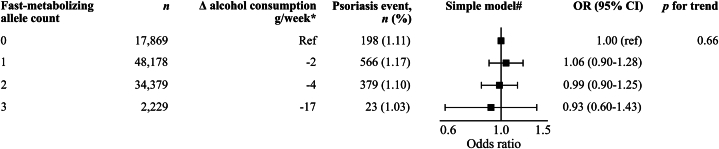
Genetic analysis. Risk of incident psoriasis according to the number of fast-metabolizing alleles in the genes *ADH1B* and *ADH1C* using logistic regression. Since genes are present from birth and thus not prone to confounding, we only used the simple model. ∗Geometric mean difference compared with the group before. #Adjusted for sex, age, study cohort and year of inclusion. *CI*, Confidence interval; *n*, number; *OR*, odds ratio.

We found no increased risk of incident psoriasis according to the number of fast-metabolizing alleles, with an odds ratio of 0.93 (96% CI 0.60-1.43) in individuals with 3 or more fast-metabolizing alleles compared to individuals with no fast-metabolizing alleles (*P* value for trend = .66) (Fig 4). Using a more restrictive definition of psoriasis gave similar results (Supplementary Fig 4, available via Mendeley at https://data.mendeley.com/datasets/xv2x88cd5g/1).

## Discussion

In this study of more than 100,000 individuals from the Danish general population we found a 30% increase in risk of incident psoriasis in those drinking >224 g alcohol/week compared to those drinking 1-112 g alcohol/week in the observational analyses. However, genetic analyses indicate that this association is not causal and might be due to bias such as confounding or reverse causation.

The observational association between alcohol consumption and psoriasis has been reported in several studies.^8^, ^9^, ^10^, ^11^ This association could be explained by the fact that alcohol has been shown to have proinflammatory effects in the skin^37^^,^^38^ as well as being involved in other comorbidities such as diabetes, hypertension, and dyslipidemia which are all thought to be risk factors of psoriasis.^1^ However, the literature on this association is conflicting with 1 large study finding an association only among beer drinkers, but not among wine or liquor drinkers.^9^ In contrast, another study found no association at all.^12^

Two previous studies have examined alcohol consumption and risk of psoriasis using MR methods, both with negative results.^39^^,^^40^ Both studies use data from the United Kingdom biobank. Thus, our study is the first to verify these results in a different cohort. Furthermore, they use summary-level data from genome wide association studies, unlike this study using individual-level data. Our genetic instrument consists only of 2 genetic variants, compared to 99 and 98 in the previous studies. However, we only use variants with a well characterized molecular mechanism for their effects on alcohol consumption. A disadvantage of including more variants with an unknown mechanism is a greater risk of bias from horizontal pleiotropy, meaning effects of the genotype on the outcome, that do not involve the exposure of interest.^41^ Our study show that even though the genetic instrument only consists of genetic variants with a known mechanism of action in order to avoid bias from horizontal pleiotropy, we still do not find evidence of a causal relationship between alcohol consumption and incident psoriasis. This interpretation is supported by the fact that the observational association we observe is not particularly strong. We find no association between moderate alcohol consumption and psoriasis, while high alcohol consumption is only associated with a 30% increase in risk. This modest effect could plausibly be explained by residual confounding, which could also explain the conflicting results in prior observational studies^8^^,^^10^^,^^12^ and why 1 study finds an effect of the type of alcohol (beer, wine, liquor) and risk of psoriasis.^9^ In the latter case, the preference for a certain type of alcohol could be influenced by factors such as socioeconomic status and demographic characteristics.

Several limitations of our study should be considered. First, there is a possibility that our genetic instrument does not have sufficient statistical power to capture the causal association between alcohol and psoriasis. However, our genetic instrument had a valid F-statistic of 38.4. Generally, an F-statistic higher than 10 is considered adequate, making the likelihood of our study being underpowered small.^26^^,^^42^ Secondly, MR assumes that the genetic instrument is not associated with confounders. We found that our instrument was associated with sex and diabetes, as seen in Table I. As psoriasis occurs evenly among men and women, sex is not a confounder of psoriasis and therefore likely not an important source of bias.^1^ The association with diabetes could be a source of bias, but if diabetes were a confounder, we would expect an overestimation of the association between the genotype and psoriasis, which cannot explain our null finding. Thirdly, in our observational study alcohol consumption is self-reported at the time of inclusion, which may differ from the alcohol consumption at the time of onset of psoriasis. However, in general alcohol consumption is somewhat stable over time.^43^^,^^44^ Furthermore, self-reported alcohol consumption may underestimate actual consumption.^45^ While this may impact our observational analyses, it does not affect the genetic analyses since alcohol consumption is not used in these analyses. Based on the self-reported data, we estimated that the difference in alcohol consumption due to the genotype was 17 g/week. If alcohol consumption was under-reported, the true difference between genotypes would be larger, but this would not alter our conclusion. Fourthly, psoriasis is identified using the DNPR, which mainly records hospital-diagnosed psoriasis, which means we have not examined the effects of alcohol on mild psoriasis. Lastly, the study was conducted only on individuals of Scandinavian descent, which may limit the generalizability of the study.

In conclusion, we found a 30% higher risk of psoriasis in individuals with an alcohol intake more than 224 g alcohol/week. However, genetic analyses indicate that this association is not causal. This finding adds to the understanding of the causal relationship between alcohol consumption and risk of incident psoriasis. However, even though we find that alcohol may not cause psoriasis, it is still recommended to avoid excessive alcohol consumption to reduce the risk of alcohol-related comorbidities.

## Conflicts of interest

Jordan, Dr Vedel-Krogh, and Bojesen report no conflicts of interest. Dr Näslund-Koch has served as an investigator for Galderma, AbbVie, LEO Pharma, Novartis, and CSL Behring. Skov has received research funding from Novartis, Bristol-Myers Squibb, AbbVie, Janssen Pharmaceuticals, the Danish National Psoriasis Foundation, the LEO Foundation and the Kgl Hofbundtmager Aage Bang Foundation and honoraria as consultant and/or speaker for AbbVie, Eli Lilly, Novartis, Pfizer, and LEO Pharma, Janssen, UCB, Almirall, Bristol-Myers Squibb, and Sanofi. She has served as an investigator for AbbVie, Pfizer, Sanofi, Janssen, Boehringer Ingelheim, AstraZeneca, Eli Lilly, Novartis, Regeneron, Galderma, and LEO Pharma.
